# Rats adaptively seek information to accommodate a lack of information

**DOI:** 10.1038/s41598-023-41717-x

**Published:** 2023-09-02

**Authors:** Shoko Yuki, Yoshio Sakurai, Dai Yanagihara

**Affiliations:** 1 Graduate School of Arts and Sciences, The University of Tokyo, 3-8-1, Komaba, Meguro-ku, Tokyo, 153-8902 Japan; 2 Graduate School of Brain Science, Doshisha University, 1-3, Tatara Miyakodani, Kyotanabe-shi, Kyoto, 610-0394 Japan

**Keywords:** Operant learning, Short-term memory

## Abstract

Metacognition is the ability to adaptively control one’s behavior by referring to one’s own cognitive processes. It is thought to contribute to learning in situations where there is insufficient information available from the environment. Information-seeking behavior is a type of metacognition in which one confirms the necessary information only when one does not have the necessary and sufficient information to accomplish a task. The rats were required to respond to a nose poke hole on one wall of the experimental box for a certain period of time and then move to the opposite side at a specific time. Unfortunately, they were unable to match the timing when responding to the hole on one side. Therefore, they had to look back and confirm that now was the right time. The results obtained by analyzing these looking-back movements using a motion capture system showed that this behavior occurred frequently and rapidly in situations of insufficient information, such as in the early stages of learning, but was hardly observed and became slower as learning progressed. These results suggest that rats can adjust their behavior in response to a lack of information more flexibly than previously assumed.

## Introduction

When cooking a dish for the first time that we have never cooked before, many of us check recipes at each step of the cooking process. However, as one becomes increasingly accustomed to cooking, this becomes less frequent, and eventually, it becomes possible to prepare food without checking for clues. This ability to monitor one's own knowledge and control one's behavior (e.g., whether to refer to a recipe) in response to the results is referred to as metacognition. Comparative metacognitive research in animals has conventionally focused on the ability to monitor internal states^1–6^. In recent years, however, there has been an increasing focus on the process of controlling behavior based on the results of such monitoring. In particular, information-seeking behavior, in which animals internally assess whether one possesses enough information to accomplish a task and seek information if it is lacking, has been extensively studied in monkeys^7–12^, rats^13,14^, and crows^15^. Studying metacognition as information-seeking behavior contributes to our functional understanding of metacognition, the mechanism that enables flexible and efficient learning by avoiding unnecessary checking. Hampton et al.^9^ conducted an experiment with Rhesus monkeys in which food was placed randomly in one of four opaque tubes, and the monkeys were asked to choose only one of the four tubes. Monkeys were rewarded with food if they were in the tubes they chose. When monkeys were not informed in advance which tube the food was hidden in, they looked into the depths of the tubes to confirm which tube the food was in and then selected the tube. In most individuals, the frequency of this peering behavior was substantially higher when the monkeys did not know where the food was hidden in comparison to when they knew where the food was. This difference in frequency between conditions suggests that Rhesus monkeys sought more information when they did not have information about the location of food.

Similar experiments were also conducted with chimpanzees, orangutans, bonobos, and gorillas^10,11^, with the same results as for Rhesus monkeys. Furthermore, when the time between hiding the food and being able to choose a tube was delayed^10^, and the quality of the reward was enhanced^11^, the frequency of looking in increased. The finding that monkeys exhibited appropriate peering behavior in such diverse situations is a strong rebuttal to the non-metacognitive interpretation of the experimental results that this behavior is governed by the characteristics of the specific experimental conditions and/or one's own behavioral biases.

Tu et al.^12^ further showed that Rhesus monkeys regulate their information-seeking behavior at the level of number of times, that is, they keep seeking until they have gathered enough information. In this experiment, the monkeys were required to classify the images on the screen into several categories; however, at the beginning of each trial, the image to be classified was obscured by several interfering stimuli. Once the monkey touched a specific icon on the screen (called the “reveal button”), one of the obstructing stimuli was removed, and part of the image that had been hidden by the stimuli became visible. If the image is completely obscured, there are no cues for categorization. Therefore, information seeking in this experiment was defined as the removal of obstructive stimuli by touching the “reveal button” until sufficient information for categorization was obtained. The experiment showed that the monkeys almost never proceeded to categorization without touching this button at all, and the more the image was hidden by obstructing stimuli, the more they pressed the button. When the classifying image was smaller and completely hidden behind one of the multiple obstructing stimuli, they pressed the button until the critical obstructing stimulus was removed.

Thus, studies with Old World monkeys have yielded some positive results, but the results of experiments with other animal species are unclear. For example, a species of New World monkey, the capuchin monkey, exhibited a high frequency of information-seeking, even when there was not much need for that, such as observing where the experimenter hid food or having only one face-down cup where food could be hidden^7,8^. Similarly, crows are known to exhibit information seeking in nearly all trials, regardless of the number of cues available to them about the location of hidden food^15^. These results suggest that in animal species other than Old World monkeys, behavior apparently akin to information-seeking was acquired mainly as a conditioned response to a specific situation where specific experimental equipment such as a tube was presented rather than as a behavioral control based on the amount of information they possess.

It has also yet to be shown that rats exhibit information-seeking behavior. Roberts et al.^13^ examined whether rats exhibited information-seeking using a spatial discrimination task in a T-maze. A visual stimulus was placed at the junctions of the paths to provide clues as to which path the reward was located at the end of. After the rats learned the correspondence between the visual stimulus and the reward path, a barrier was placed in front of the visual stimulus. In this situation, researchers assessed whether the rats would look at the visual stimulus and use the information to select a path. As a result, as the barrier became higher and the hidden area of the visual stimulus became larger, their correct response rate decreased uniformly. No results were obtained, suggesting that the rats voluntarily used visual information to select a path to run. Similarly, in the Foote and Crystal^14^ experiment, information-seeking behavior itself was associated with a small amount of immediate reward, but the rats did not exhibit significantly more information-seeking on the difficult trials.

There is a possibility that animals other than Old World monkeys do not experience information seeking because the utility level of their task is not appropriate^16^. For example, in a discrimination task using a T-maze, rats have a 50% chance of obtaining rewards, even if they randomly choose a path. Therefore, it is possible that the reward expectation increased by overcoming the barrier at the junction, and checking the visual cue was not commensurate with the cost required for the behavior. In the case of the capuchin monkeys and crows, on the other hand, the cost of looking into the tube may have been too low. Consequently, the most effective strategy might have been to examine the tubes to locate food, regardless of the amount of information they had.

Therefore, we examined whether rats exhibit information-seeking behavior, focusing on the learning process in which the utility of information acquisition is high in the early stages and then declines. Elucidating whether rodents, especially rats, exhibit information-seeking will contribute to the exploration of the adaptive significance of metacognition in mammals and, ultimately, its evolutionary origins.

The rats were required to switch responses to the other hole behind them at specific times while responding to the front nose-poke hole. Immediately after that, the hole behind them was dimly lit, and their responses were detectable. Thus, the rats were expected to look back and confirm until they learned when the back hole was lit. Conversely, once they learn the timing, they will not interrupt their response to the front hole with unnecessary confirmation. To measure this look-back for confirmation, their behavior during the task was measured with a high-speed camera and analyzed. If this behavior is intended to compensate for the lack of information, it should occur at a high frequency in situations where information is insufficient. This is typically observed in the early stages of learning, and becomes more frequent as time passes from the start of the trial, but should become less frequent as learning progresses. Additionally, the less information they have about when the lights behind them will turn on and the greater the uncertainty, the faster they will look back.

## Methods

### Animals

Six adult male Long-Evans rats were used in this experiment. All the rats were at least 10 weeks old at the start of training. During the experiment, the rats maintained at least 80% of their body weight prior to the experiment. This experiment was reviewed and approved by the Ethics Committee for Animal Experiments at the University of Tokyo. All methods were carried out in accordance with the relevant guidelines and regulations and were reported in accordance with ARRIVE guidelines (https://arriveguidelines.org).

### Apparatus

The experiment was conducted in an operant chamber (H: 45 cm × W: 33 cm × D: 30 cm; O’Hara Co. Ltd., Tokyo, Japan) located inside a soundproof box (Japan Shielded Enclosures Co. Ltd., Tokyo, Japan). The equipment in the operant chamber was controlled, and responses were recorded using an Arduino Mega 2560 (https://www.arduino.cc). One nose poke hole was placed in the center of each of the two opposing sides of the operant chamber. The nose poke holes were illuminated from the inside using LED lights, and the brightness was controlled by the height of the voltage supplied to them. An infrared sensor was placed inside these holes to detect whether the rats had inserted their noses into them. One of these holes also had a water supply nozzle through which water rewards were presented.

### Tasks

**Figure 1:**
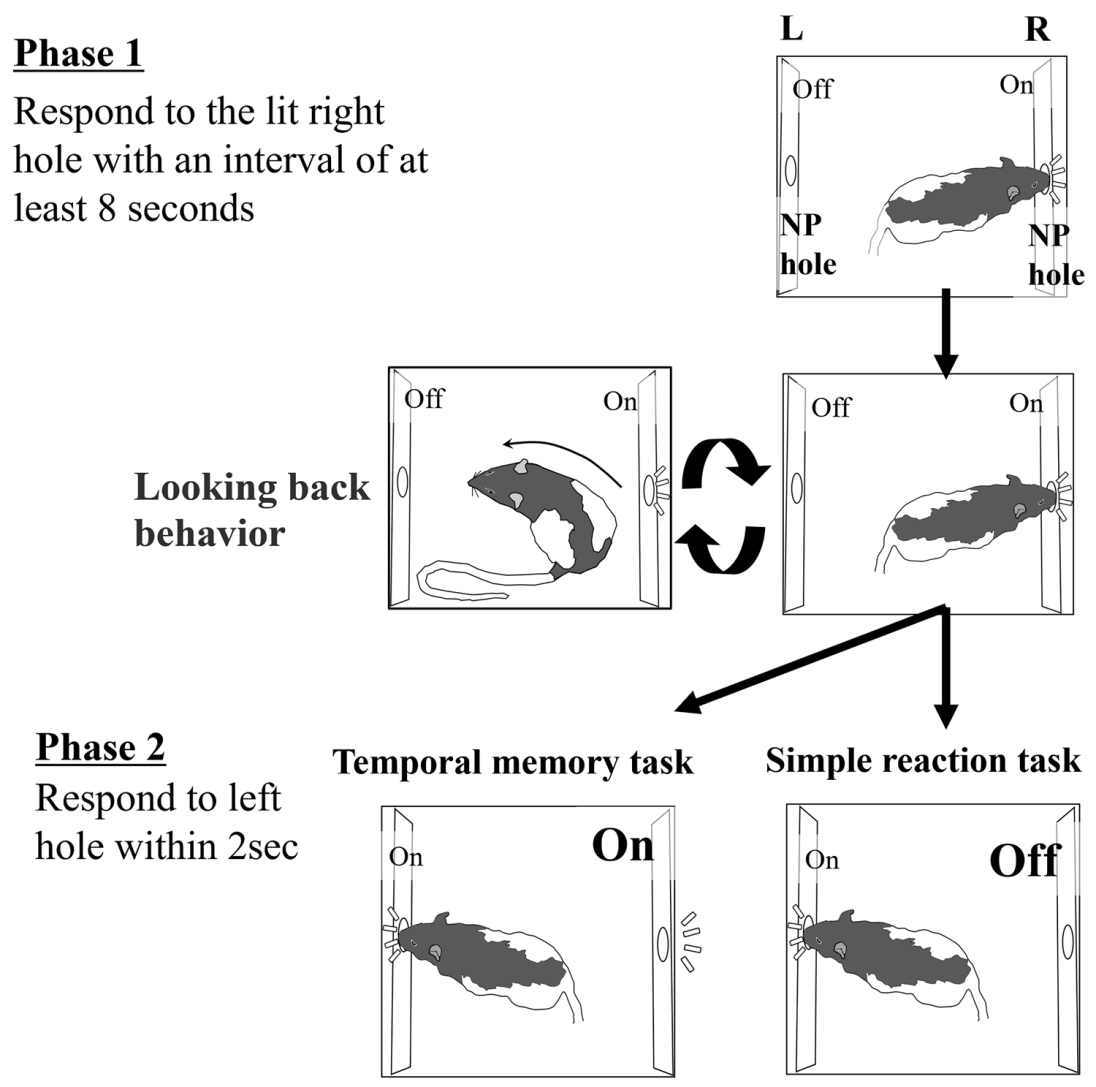
Schematic diagram of the experimental tasks. Phase 1 was identical for both the temporal memory task and the simple reaction task. In the former task, the right-side hole remained lit even when the left side was lit in Phase 2, while in the latter, the right side was turned off at that time.

In this experiment, each rat performed both a temporal memory task to investigate information-seeking and a simple reaction task as a control task (Fig. 1). Rats were divided into two groups of three rats each. One group first completed a temporal memory task, followed by a simple reaction task. The other group performed the simple reaction task first, followed by the temporal memory task.

In the temporal memory task, one trial was initiated by illuminating the right nose poke hole. Once the rat responded to that hole, the timer started counting, and if the rat responded again after 8 s had elapsed from the first response, the left-side hole was dimly lit for 2 s, while the right-side hole remained lit. The rats were rewarded with water (4 µl × 9 drops) for responding to the left hole while it was lit. The brightness of the LED light in the left hole was reduced by suppressing the voltage applied to the light to 10% (0.5 V) of that applied on the right side (5 V). This was to prevent reflected light from passing through the operant box. That is, the design required the rats to look back to confirm that the left hole was lit. Meanwhile, since look-back behavior did not contribute to terminating Phase 1 by itself, it was rather adaptive not to perform this once the rats had learned the end timing of Phase 1.

In the simple reaction task, the sequence of behaviors required by the rats was the same as in the temporal memory task. However, in this task, the rats did not need to check the left-side light at all because the left-side light was turned on at 100% brightness and the right-side light was turned off at the end of Phase 1. In both tasks, the inter-trial interval (ITI) was set at least 18 s. To avoid waiting in front of the right hole or continuing to respond to this hole during the ITI, the next trial was initiated only if there was no response to the right hole during the last 3 s of the ITI. If there was a response during the last 3 s, the ITI was extended by 3 s each until there was no response for 3 s.

### Training & test protocols

Prior to the experiment, the rats were habituated to the operant box and the water supply nozzle. Then, shaping was performed sequentially, starting with responses to the illuminated left hole. Eventually, the rats learned to obtain rewards by responding within 2 s to the lit left hole and by re-reacting 0.5, 2, and 4 s after the initial response to the lit right hole. Training was conducted once a day for two hours in one session, and the rats were transferred to the test after they were confirmed to be able to perform more than 800 trials in four sessions for each of the 0.5-, 2-, and 4-s time intervals for Phase 1. The group that first underwent the temporal memory task was then retrained in the simple reaction task and confirmed if they met the same achievement criteria. The other group received retraining in reverse order.

The test was conducted in four sessions with a time interval of 8 s for Phase 1. One rat that was able to perform fewer trials per session in the temporal memory tests had two additional sessions for a total of six sessions. The number of seconds Phase 1 lasted in each trial was recorded using the Arduino—millis() function. All trials that satisfied any of the following conditions were excluded from the subsequent analyses: The first trial could not reach the left hole after terminating Phase 1 during the 12-s recording period. Next was a trial in which the ITI from the previous trial deviated from the mean by more than ± 3 SD. The last trial was one in which the measured behavioral indices deviated from the mean by more than ± 3 SD, as in the second case. Finally, the data up to the 550th trial that met the above conditions for all individuals was used for subsequent analyses.

### Video recordings and analysis

Behaviors during Phase 1 were recorded using a high-speed camera (HAS-U1, DITECT Corporation, Tokyo, Japan) at 150 fps for 12 s (1800 frames in total). The video recording started automatically when the rat started the trial by responding to the lit right hole, ended after 12 s, and was automatically saved.

**Figure 2:**
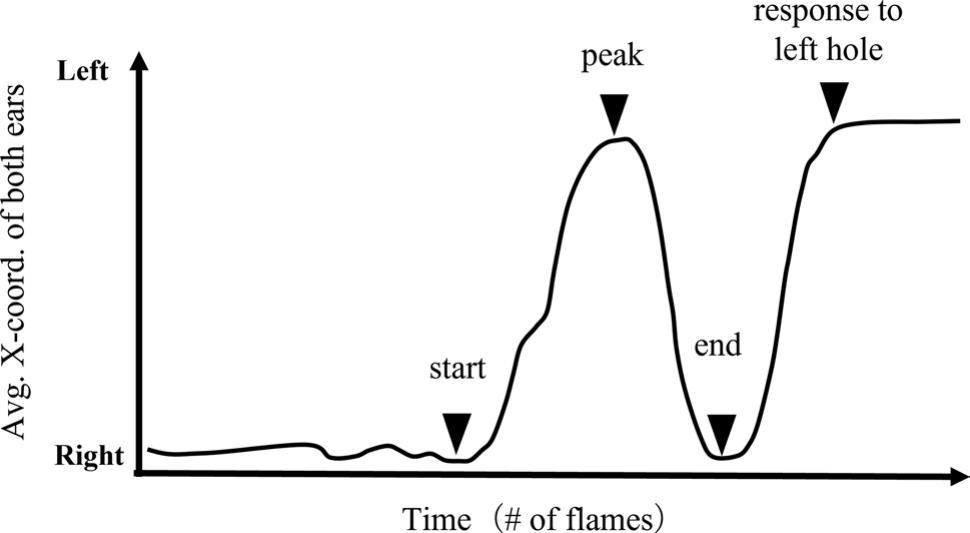
Schematic diagram of the horizontal movement of the rat in Phase 1. The look-back behavior was defined as a curve in the X-coordinate caused by moving once from right to left and then back to right again during Phase 1. The timing was defined as the number of frames at the time of the most leftward look-back (peak), and the velocity was expressed as the change in the X-coordinate from start to peak divided by the change in the number of frames.

The behavior of the rats during the task can be represented mainly as a right-to-left movement on the X-axis, and look-back behavior was defined as a right-to-left head movement followed by a left-to-right head movement. We used DeepLabCut (ver. 2.2.0.6)^17^ to extract the two-dimensional coordinates of the bilateral ears of the rats from the video. The averaged values of these X-coordinates were regarded as a representative value of their head position and were used to calculate the horizontal travel distance and detect look-back behavior. The travel distance was defined as the sum of the absolute values of the differences in the X-coordinates between adjacent frames during Phase 1. Look-back behavior was detected through the “islocalmax” function of MATLAB 2022a (Natick, Massachusetts: The MathWorks Inc.) as positive and negative local maximum detection when the left of the X-coordinate was positive, and the right was negative (Fig. 2). The timing of the negative local maximum immediately preceding each positive local maximum (peak of look-back) was defined as the start of the look-back, and the negative local maximum immediately following the positive local maximum was defined as the end of the look-back. The data used for detection were the results of applying a 25th-order 3 Hz low-pass FIR filter to the raw data.

To exclude backward head movements that were not look-back, the prominence parameter, an option of the function (https://www.mathworks.com/help/signal/ug/prominence.html), was set to 50 and 5 for positive and negative local maximum detections, respectively. The amplitude of the look-back was defined as the difference between the X-coordinate of the peak of the look-back and the greater value of the X-coordinate of either the start or end of the look-back. Peaks with an amplitude of less than 50 pixels were not considered look-back behaviors. The number of frames where the peak was detected in Phase 1 of each trial was used as an indicator of when the look-back occurred, and the increment of the X-coordinate from start to peak divided by the increment of the number of frames was used as an index of velocity.

### Predictions

If the rats performed look-back behavior to gather the information needed to perform the task, then the frequency should change according to the need for the information. In other words, the utility of looking back to the left side during Phase 1 is expected to be higher in a temporal memory task, because it is difficult to determine when Phase 1 has ended if the rats were responding to the right side. Therefore, a more frequent look-back would occur in the temporal memory task than in the simple reaction task, where the end of Phase 1 is clearly indicated by the turning off of the light on the right side. Within the temporal memory task, although the look-back will initially occur at a high frequency according to the elapsed time of Phase 1, the frequency will decrease with each trial, and the timing bias according to the elapsed time will disappear. Furthermore, if look-back is driven by the need for confirmation due to uncertainty in the timing of the end of Phase 1, then the speed should be faster in the temporal memory task with increased uncertainty than in the simple response task and slower as the number of trials increases and uncertainty decreases.

### Statistical analysis

In this study, the duration of Phase 1 and the total distance traveled in the horizontal direction during Phase 1 as indices of the rats' learning process during the task, and the frequency and velocity as indices of look-back behavior were included as dependent variables in the statistical analysis. To correctly estimate the effects of the independent variables while accounting for individual differences, we used general linear mixed models (GLMMs) with the MATLAB “fitglme” function. The dependent variables were assumed to follow a Poisson distribution for the number of lookbacks, and a Gaussian distribution for the rest. In common with all analyses, a logarithm was used as the link function and dummy variables for the individual. The order in which the two tasks were performed was included as a random intercept.

As fixed effects, the number of trials, tasks, and their interactions were included in the model as independent variables. This was done when the dependent variables were Phase 1 duration and total horizontal travel distance, and the analyses were conducted at the trial level. When the frequency and velocity of look-backs were included as dependent variables, the number of trials, tasks, timing of look-backs, and all combinations of their interactions were included as independent variables. In these analyses, only the data from the beginning of Phase 1 to 1250 frames (corresponding to 8.3 s from the start) were analyzed. For the number of trials and timing, raw values were not directly used as independent variables, but the number of bins was used as an independent variable by discretizing the first 1250 frames of each Phase 1 of 550 trials into bins of 50 trials, 50 frames each, in two dimensions. The dependent variables were also the aggregated number of occurrences of lookback and their average velocity in each bin.

## Results

**Figure 3:**
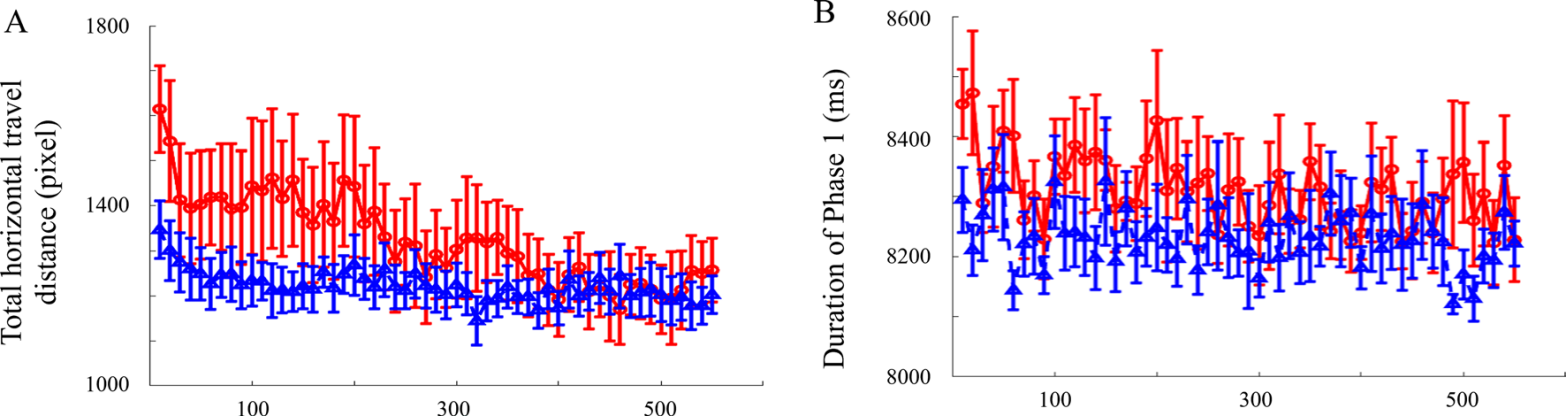
The change in (**A**) total horizontal travel distance during Phase 1 and (**B**) duration of Phase 1 in the temporal memory (red) and simple reaction (blue) tasks. Each measurement was averaged every 10 trials for visualization, and error bars represent standard errors.

As the rats learned the task, they should have changed their behavior to minimize the amount of horizontal travel and terminated Phase 1 as soon as possible. Therefore, we analyzed the changes in the total distance traveled and Phase 1 duration as the number of trials progressed using GLMM (Fig. 3; Table 1). The results showed that the total distance traveled decreased significantly with the number of trials (p < 0.001) and was significantly shorter in the simple reaction task (p < 0.001). The decay of the total distance traveled with respect to the number of trials was slower in the simple reaction task (p < 0.001). Similarly, the Phase 1 duration also decreased significantly with the number of trials (p < 0.001) and was substantially shorter in the simple reaction task (p < 0.001). These results suggest that the rats exhibited progressive behavioral adaptations during the tasks and that more conservative behavior was achieved in the simple reaction task.

**Table 1:** Regression coefficients of the GLMM model for total horizontal travel distance and duration of Phase 1.

Variables	β	Standard error	t-value	Degree of freedom	p-value	95% Confidence intervals	
						Lower	Upper
1. Total horizontal travel distance (pixel)							
Intercept	7.3	0.064	114.47	6439	0	7.17	7.42
# of trials	− 0.00043	1.54E−05	− 28.24	6439	1.48E−165	− 0.00046	− 0.0004
Task [simple reaction task]	-0.17	0.007	− 23.95	6439	1.55E−121	− 0.18	− 0.15
# of trials × Task	0.00032	2.27E−05	14.2	6439	4.49E−45	0.00028	0.00037
2. Duration of phase 1 (ms)							
Intercept	9.03	0.0067	1349.8	6435	0	9.02	9.04
# of trials	− 2.20E−05	4.60E−06	− 4.81	6435	1.53E−06	− 3.11.e−05	− 1.31.e−05
Task [simple reaction task]	− 0.013	0.0021	− 6.28	6435	3.66E−10	− 0.017	− 0.009
# of trials×Task	1.30E−05	6.50E−06	1.95	6435	0.052	− 9.94.e−08	2.55.e−05

**Figure 4:**
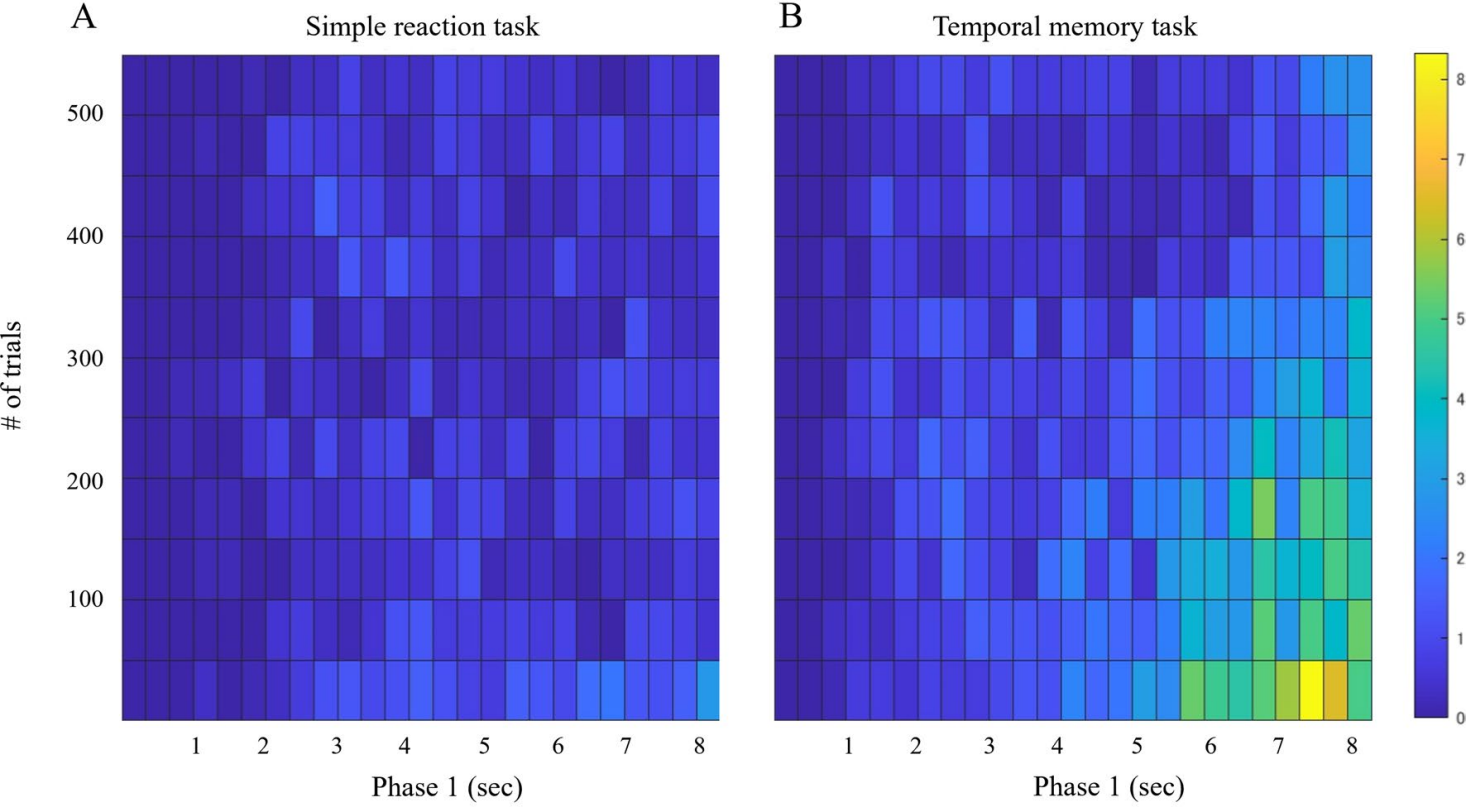
2D histogram representing the distribution of the number of look-backs in the space of elapsed time in Phase 1 (X-axis) and the number of elapsed trials (Y-axis) in (**A**) simple reaction task and (**B**) temporal memory task. The width of each bin is 50 flames and 50 trials each, and the different colors of each bin correspond to the average number of look-backs observed within that range across individuals.

On average, 1.05 (± 2.1 as one standard deviation) and 0.35 (± 0.95) look-backs were observed in 550 trials for the temporal memory task and the simple reaction task, respectively. The 2-D distribution of the frequency of look-backs is shown in Fig. 4, and the results of the GLMM analysis are shown in Table 2. The simple reaction task had significantly fewer look-backs (p < 0.001). There was a consistent pattern in both tasks; they became more frequent as the end of the first phase approached (p < 0.001) but decreased as the number of trials increased (p < 0.01). In addition, the concentration of look-back near the end of Phase 1 was weaker as the number of trials increased (p < 0.001). In the simple reaction task, however, look-back was less likely to be concentrated near the end than in the temporal memory task (p < 0.001). The effect of increasing trial number itself (p = 0.14) and its interaction with elapsed Phase 1 time (p = 0.75) did not differ significantly from the temporal memory task.

**Table 2:** Regression coefficients of the GLMM model for look-back frequency.

Variables	β	Standard error	t-value	Degree of freedom	p-value	95% confidence intervals	
						Lower	Upper
Intercept	− 1.27	0.48	− 2.64	3292	0.0082	− 2.21	− 0.33
# of trial bins	− 0.06	0.021	− 2.93	3292	0.0035	− 0.1	− 0.02
# of flame bins in Phase 1	0.13	0.007	19.95	3292	1.10E−83	0.12	0.14
Task [simple reaction task]	− 0.79	0.23	− 3.46	3292	0.00055	− 1.23	− 0.34
# of flame bins × # of trials	− 0	0.001	− 3.83	3292	0.00013	− 0.01	− 0
# of trials × Task	0.053	0.036	1.47	3292	0.14	− 0.02	0.12
# of flame bins × Task	− 0.05	0.012	− 3.85	3292	0.00012	− 0.07	− 0.02
# of flame bins **× **# of trial bins **× **Task	6E−04	0.002	0.32	3292	0.75	− 0	0.005

**Figure 5:**
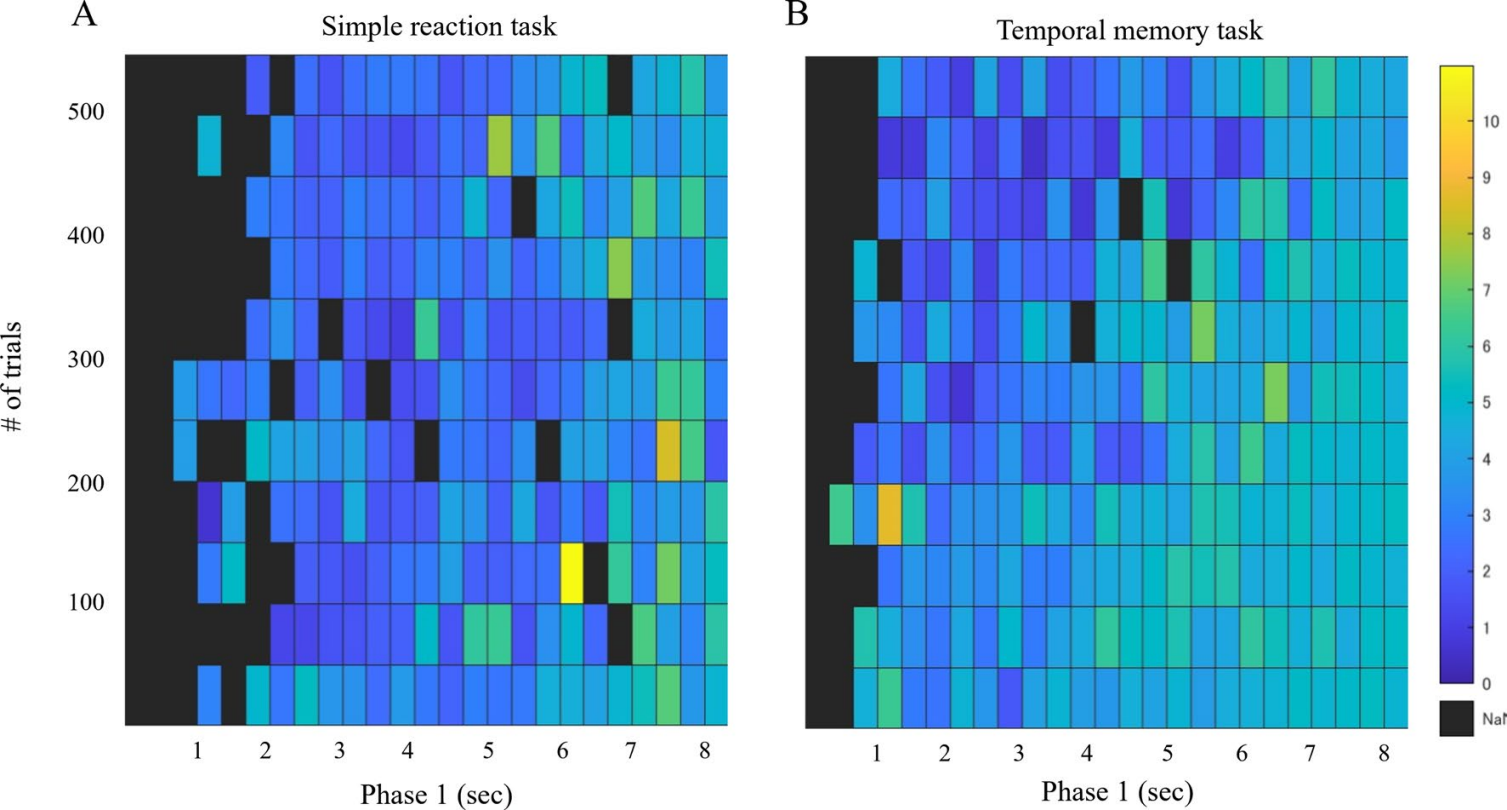
2D histogram representing the distribution of the velocity of look-backs in the space of elapsed time in Phase 1 (X-axis) and the number of elapsed trials (Y-axis) in (**A**) simple reaction task and (**B**) temporal memory task. The different colors of each bin correspond to the averaged velocity.

The 2-D distribution of the look-back velocity is shown in Fig. 5, and the results of the GLMM analysis are presented in Table 3. The velocity of look-back was consistently higher in the temporal memory task (p < 0.001). It also slowed down as the number of trials increased (p < 0.001). On the other hand, it became faster as the end timing approached (p < 0.01), and this became stronger as the number of trials increased (p < 0.01). In the simple reaction task, velocity did not decrease with the number of trials (p = 0.027) and became faster as the end timing approached (p < 0.001), compared to the temporal memory task. It also suggested that as the end of Phase 1 approached, the velocity tended to slow as the number of trials increased. (p = 0.012).

**Table 3:** Regression coefficients of the GLMM model for look-back velocity.

Variables	β	Standard error	t-value	Degree of freedom	p-value	95% confidence intervals	
						Lower	Upper
Intercept	1.325	0.24	5.56	1153	3.28E−08	0.86	1.79
# of trial bins	− 0.04	0.009	− 4.42	1153	1.09E−05	− 0.06	− 0.02
# of flame bins in Phase 1	0.008	0.003	3.01	1153	0.0026	0.003	0.014
Task [simple reaction task]	− 0.43	0.11	− 3.93	1153	8.96E−05	− 0.64	− 0.22
# of flame bins × # of trial bins	0.002	5E−04	3.2	1153	0.0014	6E−04	0.002
# of trial bins × Task	0.042	0.019	2.22	1153	0.027	0.005	0.08
# of flame bins × Task	0.019	0.006	3.42	1153	0.00065	0.008	0.03
# of flame bins **× **# of trial bins **× **Task	− 0	0.001	− 2.52	1153	0.012	− 0	− 0

## Discussion

The purpose of this study was to examine whether rats selectively collect the information they need when they do not have enough information to accomplish their task, similar to Old World monkeys. Rats were subjected to two types of tasks: a temporal memory task in which they had to look behind themselves to obtain the information necessary to perform the task, and a simple reaction task in which they could obtain the necessary information without such an extra action. In both tasks, rats were observed looking back. We hypothesized that this behavior is a demonstration of information seeking in rats and tested this hypothesis based on task differences and between-trial changes in the frequency, timing, and velocity of this behavior.

The experimental results showed that the frequency of look-back was much higher in the temporal memory task than in the simple reaction task. Furthermore, in the temporal memory task, the frequency of look-back occurred at a high frequency according to the elapsed time of Phase 1 immediately after the start of the task, but the frequency decreased as the number of trials increased, and the response bias according to elapsed time also decreased. Regarding velocity, it was found that while it was faster in the temporal memory task than in the simple reaction task, it slowed down as the number of trials increased. This reduction in velocity over the trials did not occur during the simple reaction task. These results suggest that look-back is information-seeking based on the degree of information insufficiency at the time of switching behavior.

In comparative studies of metacognition, the possibility that the obtained results are only apparently metacognitive and can be explained by lower-order interpretations needs to be fully discussed^18–20^. In particular, it is often argued that seemingly metacognitive behaviors that emerge in studies that focus on monitoring processes, such as confidence-based wagering or task avoidance paradigms, can be explained in terms of mere associative learning^21,22^. The reason is that in such a paradigm, behaviors that are presumed to be the reflection of metacognition are directly conditioned. Although not directly conditioned, information-seeking behavior in our experiment affects the efficiency of reward attainment, and its confounding possibilities must be discussed.

A possible associative interpretation of the look-back behavior in the temporal memory task is that it was a hasty behavioral switch to get the water reward, which was gradually erased by associative learning by not lighting the left nose poke hole (and turning off the right nose poke hole only in the simple reaction task). This associative learning would explain all the trial-to-trial changes in look-back behavior in the simple reaction task, where there is no lack of information about when to look back. On the other hand, two factors—this associative learning and the reduction of information insufficiency due to learning progression—would have influenced the trial-to-trial changes in look-back behavior in the temporal memory task. Thus, associative learning could explain results common to both tasks, such as the fact that the overall frequency of look-back decreased with the number of trials, but the frequency rose with the elapsed time of Phase 1 as the number of trials increased. However, the differences between the tasks would not be explained by associative learning alone. Specifically, look-backs were generally more frequent and faster in the temporal memory task, and frequency and deceleration increased more with elapsed time in Phase 1 with successive trials than in the simple response task. These results suggest that the look-back behaviors observed in the two tasks have different characteristics. Alternatively, the look-back behavior in the temporal memory task could be interpreted as merely a manifestation of the animal’s innate tendency to randomly explore and then automatically repeat until a reward is obtained, resulting in a superficial appearance of information seeking^23^. However, this factor alone would not explain the look-back behavior in the temporal memory task. In a completely random scenario, the behavior would be observed throughout Phase 1, but it became pronounced at the end.

Finally, we address the change in the velocity of look-back behavior in the temporal memory task. We expected look-back to slow down as the number of trials increased and uncertainty decreased, if look-back was driven by uncertainty about the timing of the end of Phase 1. However, the degree of acceleration with elapsed time in Phase 1 was shown to increase, even though the overall velocity in Phase 1 slowed as the number of trials increased. Perhaps this could be interpreted as a manifestation of metacognition, a reflection that the rats have learned when to look back due to uncertainty. This is because we did not observe this tendency in the control task. Likewise, we also cannot completely exclude the possibility of a low-level interpretation, in which looking back was driven by anticipation of reward, which increased over time in Phase 1. The possibility that lower-order factors other than metacognition may have influenced the results does not immediately rule out the involvement of metacognition^20^. Further research is needed, however, to clearly differentiate the factors influencing the results of this experimental paradigm and how each has contributed.

The superiority of the rat as a model species for research on higher cognitive functions, where tasks tend to be quite complex, lies in the diversity of applicable experimental approaches for measuring and manipulating neural activity, and the accessibility of these methods compared to those of monkeys. Furthermore, in terms of suitability for measuring neural activity, information-seeking behavior has the advantage of being flexible in the frequency and timing of its occurrence in a single trial, in contrast to wagering or avoidance, which occur only once at a specific time in each trial. Thus, the neural correlates of metacognition can be investigated at a higher resolution. Therefore, a focus on information-seeking behavior in rats would be a breakthrough in research into the neural basis of metacognition.

## Data Availability

The datasets analyzed in the current study are available from the corresponding author upon reasonable request.
